# Inositol hexaphosphate-induced cellular response in myeloid leukemia cells is mediated by nicotinamide adenine dinucleotide phosphate oxidase activation

**DOI:** 10.20407/fmj.2018-020

**Published:** 2019-09-25

**Authors:** Asuka Kato, Yuki Hirakawa, Wakako Hiraoka

**Affiliations:** Department of Physics, School of Science and Technology, Meiji University, Kawasaki, Kanagawa, Japan

**Keywords:** Inositol hexaphosphate, Reactive oxygen species, NADPH oxidase, X-CGD cells, PLB-985 cells

## Abstract

**Objectives:**

The objective of this study was to identify the role of reactive oxygen species (ROS) generated by nicotinamide adenine dinucleotide phosphate (NADPH) oxidase, in inositol hexaphosphate (IP6)-induced metabolic disruption in human leukemia PLB-985 cells.

**Methods:**

PLB-985 and X chromosome linked *gp91-phox* gene knockout (X-CGD) cells were treated with 5, 10, or 20 mM IP6 for 24 to 72 h. Cell growth was assayed using a highly water-soluble tetrazolium salt. The rate of apoptotic and necrotic cell death was determined with an Annexin V-fluorescein isothiocyanate/propidium iodide kit. The expression of CD11b as a marker of monocytic property and of LC3 as an autophagy marker was tested, using flow cytometry combined with fluorescent antibodies.

**Results:**

Treatment with 5 and 10 mM IP6 for 24 h was found to suppress the growth of both cell lines, though the effect was more dramatic in PLB-985 cells. After 6-h treatment with 20 mM IP6, the necrosis rate of PLB-985 cells was significantly greater than that of X-CGD cells. Further, after 72-h treatment with 10 mM IP6, CD11b expression was observed in PLB-985 cells but inhibited in X-CGD cells. Autophagy monitoring after 6-h treatment with 10 mM IP6 revealed that LC3 expression was suppressed in PLB-985 cells, whereas it was somewhat increased in X-CGD cells.

**Conclusions:**

Our results suggest that NADPH oxidase activation mediates IP6-induced metabolic disruption associated with necrosis, differentiation, cell growth, and autophagy in PLB-985 cells.

## Introduction

Reactive oxygen species (ROS) comprise a group of highly reactive, oxygen-containing compounds. These compounds occur in living cells and tissues under both physiological conditions (such as during respiratory metabolism) and pathological conditions. Several exogenous sources such as carcinogens, ultraviolet light, and ionizing radiation may induce ROS formation.^1,2^ Endogenous ROS are known to play a beneficial role in host defense, cell signaling, and biosynthetic reactions.^1,2^ To maintain homeostasis of redox status, the effects of ROS are mediated by intrinsic defense mechanisms and dietary antioxidants such as vitamin C, vitamin E, glutathione, catalase, myeloperoxidase, and superoxide dismutase.^1,3^ Impaired regulation may lead to dramatic increases in ROS levels, resulting in protein damage, membrane peroxidation, and DNA cleavage.^4,5^ Cell damage that is induced by ROS is believed to be a major factor in the processes of aging, chronic inflammation, arteriosclerosis, and carcinogenesis.^6,7^

Inositol hexaphosphate (IP6, also known as phytic acid) is the phosphate ester of inositol in which six hydroxyl groups are replaced by phosphate.^8^ This compound was first identified as an important nutrient and the principal storage form of phosphorus in many plant tissues, especially bran and seeds.^9^ Grains and beans contain approximately 1%–5% IP6 by weight, which is hydrolyzed and consumed during germination for phosphate metabolism in plants.^10^ Mammalian cells and tissues are known to contain IP6 and its metabolites.^11^ Endogenous IP6, lower inositol phosphates, and inositol pyrophosphates have been shown to be involved in a wide range of biological and physiological functions such as energy storage, cation transport, signal transduction, cell proliferation, DNA repair, and mRNA export.^12–15^ The strong affinity of IP6 toward metal cations is due to the presence of the negatively charged phosphate residue,^1^ and is crucial for one of the major biological functions of IP6—its role as an antioxidant, which involves the elimination of free metals and suppression of metal-induced ROS.^16,17^ Dietary intake of IP6-rich and high-fiber ingredients is considered effective against gastrointestinal oxidative damage, which can lead to inflammation and cancer.^18^ Recent studies have investigated the potential clinical efficacy of IP6 against intestine, breast, and lung cancer.^19–22^ A review of the literature revealed that the metal-binding properties of IP6 may inhibit dental plaque formation and protect against renal calculi formation.^23,24^ In addition, IP6 has been used as a key material in the production of chelate-setting calcium biocement.^25,26^

Nicotinamide adenine dinucleotide phosphate (NADPH) oxidase, a membrane-bound enzyme complex, catalyzes the production of the superoxide free radical by transferring one electron to oxygen from NADPH during the respiratory burst of phagosomes. In addition, its isoforms play a catalytic role in various tissues.^27,28^ In a previous study, the X-linked chronic granulomatous disease (X-CGD) cell line was generated by disrupting the *gp91-phox* gene, which encodes a heme-binding membrane glycoprotein of cytochrome b558 of the human NADPH oxidase, through homologous recombination in the human leukemia cell line PLB-985.^29^ Therefore, the X-CGD cell line provides an excellent cell-based system for investigating the potential functions of NADPH oxidase and reactive oxygen intermediates.^30^ The aim of the present study was to identify the role of NADPH oxidase activation in IP6-induced metabolic disruption in leukemia cells.

## Methods

### Cell culture

The human myelomonoblastic (PLB-985) cell line was originally obtained from Dr. P. Newburger^31^; X-CGD cells prepared from PLB-985 cells through disruption of the X chromosome–linked *gp91-phox* gene were kindly gifted from Dr. S.J. Chanock.^29^ Cells were maintained in RPMI-1640 medium (Nissui Pharmaceutical Co., Ltd., Tokyo, Japan) supplemented with 10% fetal bovine serum (FBS) at 37°C in 5% CO_2_.

### Inositol hexaphosphate treatment

Inositol hexaphosphate was purchased from Sigma-Aldrich (St. Louis, MO, USA). It was directly dissolved in RPMI 1640 medium containing 10% FBS, and the pH adjusted at 7.0 using 1 M NaOH and 1 M HCl to prepare 40 mM IP6 fresh stock solution. The stock solution was added into the culture medium in which cells were being cultured, to obtain the final concentration of IP6.

### Cell growth

Cells were incubated with IP6 in non-treated 96-well plates (5×10^5^ cells/mL) for 24 h. Cell growth was assayed using a highly water-soluble tetrazolium salt (WST-8)-based proliferation assay (Dojindo Molecular Technologies Inc., Kumamoto, Japan), according to the manufacturer’s instructions. Cells were incubated with WST-8 for 4 h, after which calorimetric analysis was performed to assess the growth rate. To this end, the absorbance of WST-8 formazan was determined at 450 nm using a microplate reader (MTP-301, Hitachi High-Technologies Corporation, Tokyo, Japan). Cell growth rate was calculated by dividing the absorbance value of treated cells by the absorbance value of control cells. This was carried out for each concentration of IP6 that was tested.

### Apoptosis and necrosis

The pattern of cell death was evaluated using an Annexin V-fluorescein isothiocyanate (FITC)/propidium iodide (PI) assay (Apoptosis Detection Kit; BioVision Technologies, Inc., PA, USA). The cells (5×10^5^ cells/mL) were incubated with IP6 in an untreated flask for 6 or 24 h. After incubation, the stained cells were collected and analyzed using a Beckman FC500 flow cytometer (Beckman Coulter, Inc., CA, USA). The dot plot profile of flow cytometry using FITC and PI was used to discriminate between apoptotic (FITC+/PI–) and necrotic cells (FITC–/PI+).

### CD11b expression

Cell differentiation was assessed by analyzing CD11b (Mac1-α)—a marker of monocytic property in differentiated PLB-985 cells. Cells were treated with IP6 in a tissue culture-treated flask. After a 72-h incubation, cells were collected by centrifugation at 1,000 g for 5 min at 4 °C. After two washes with phosphate-buffered saline (PBS(–)), the cells (1×10^6^) were resuspended in 1.0 mL of PBS(–) containing monoclonal anti-human CD11b (Mac1-α)-monoclonal antibody FITC conjugate (Sigma-Aldrich). Following a 30-min incubation at room temperature (RT), the cells were analyzed using flow cytometry.

### Autophagy

To investigate the effects of IP6 on autophagy in PLB-985 and X-CGD cells, LC3 expression was measured as an autophagy marker. After a 6-h incubation with IP6, the cells were collected by centrifugation at 1,000 g for 5 min at 4°C. After two washes with PBS(–), cells were fixed in 2% paraformaldehyde/PBS(–) for 30 min at RT. Following two washes with PBS(–), the cells were permeabilized with 100 μg/ml digitonin (Tokyo Chemical Industry Co., LTD., Tokyo, Japan), for 15 min at RT. After two washes with PBS(–), the cells were incubated with rabbit anti-human LC3 polyclonal antibody (MBL; Medical & Biological Laboratories Co., Ltd., Nagoya, Japan) for 30 min at RT. Then, cells were washed with PBS(–) and incubated with a secondary donkey anti-rabbit IgG (H+L chain) polyclonal antibody FITC (MBL) for 15 min at RT in dark. After washing with PBS(–), cells were analyzed using a flow cytometer. The X-mean (average value for events in the population) was calculated using Beckman Software CXP version 2.2.

### Statistical analysis

Statistical analyses were performed on triplicate data using the unpaired Student’s *t*-test, and data are presented as standard deviation values of the mean. Statistical significance was accepted at *P*<0.05.

## Results

### Effect of inositol hexaphosphate on cell growth

**Figure 1:**
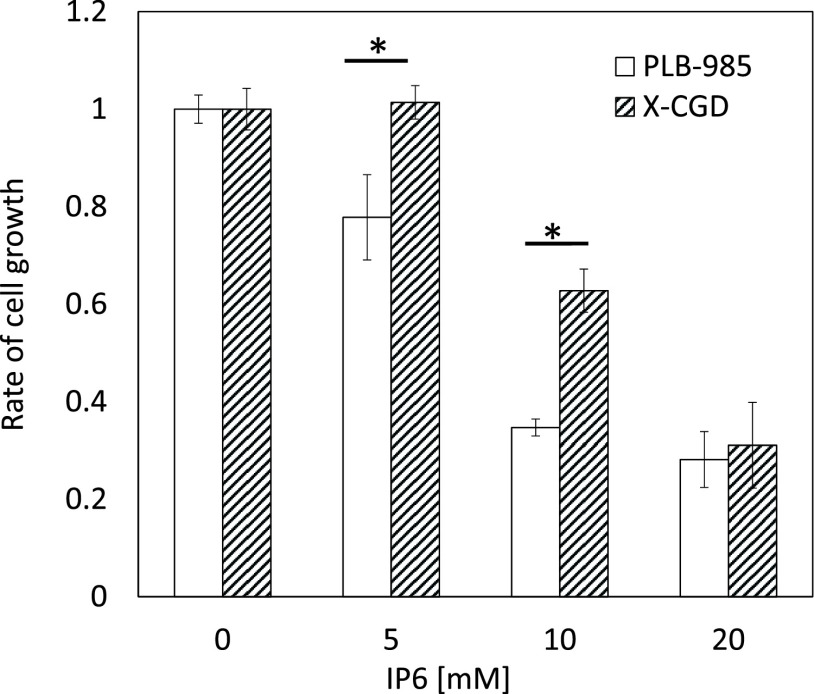
Effect of inositol hexaphosphate on the growth of PLB-985 and X chromosome linked gp91-phox gene knockout cells. Cells were incubated with inositol hexaphosphate for 24 h. Cell growth rate was assayed using a highly water-soluble tetrazolium salt, WST-8. Mean (±standard deviation) values from three independent experiments are presented. *P<0.05 (Student’s *t*-test).

Figure 1 shows the effect of IP6 treatment on cell growth. The growth of both cell lines was suppressed following treatment with 5 or 10 mM IP6, although this suppression was more dramatic in PLB-985 cells. Following treatment with 20 mM IP6, no cell growth was observed in either cell line.

### Inositol hexaphosphate-induced cell death

**Figure 2:**
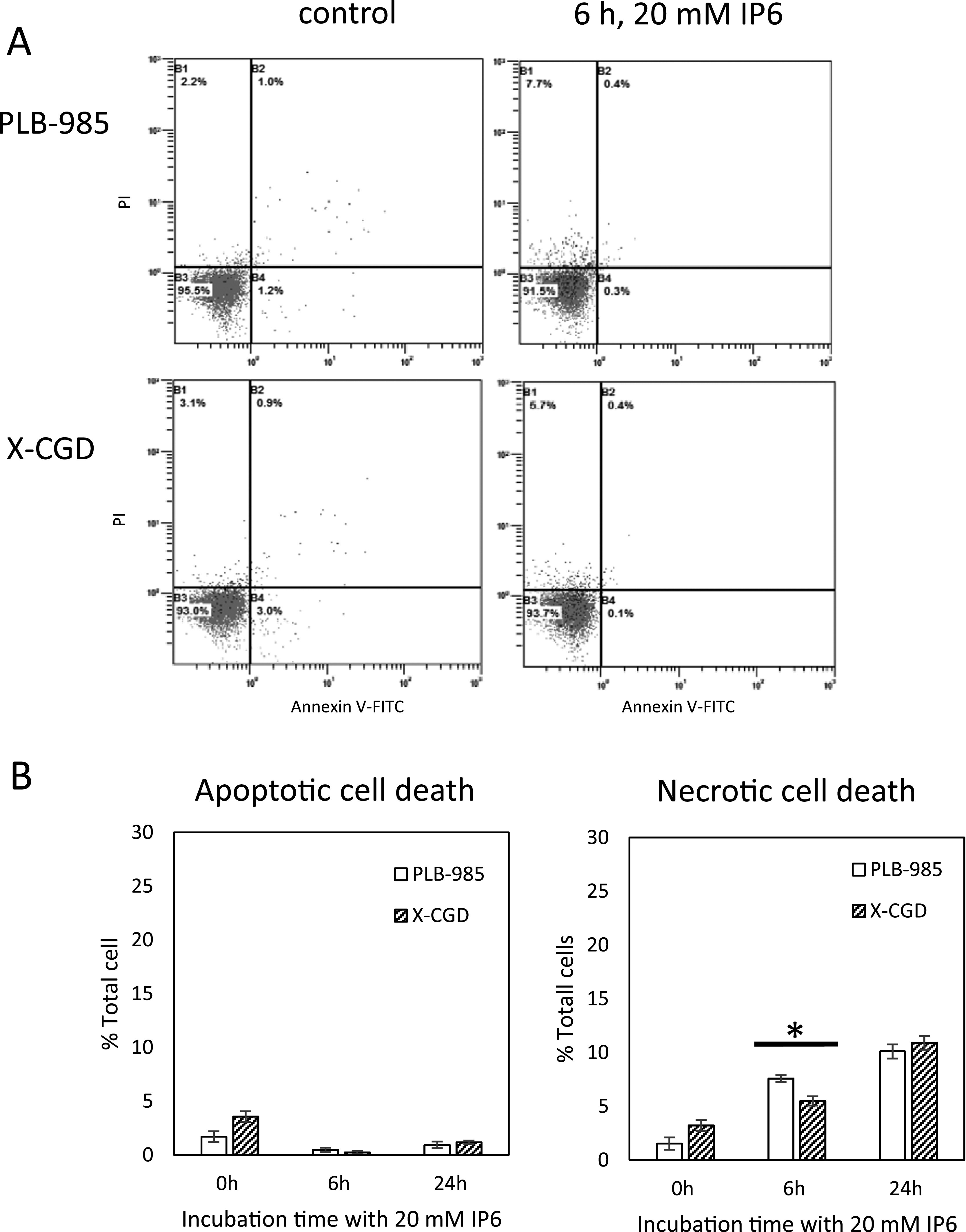
Inositol hexaphosphate -induced cell death Inositol hexaphosphate (IP6)-treated cells were analyzed with flow cytometry using Annexin V-Fluorescein isothiocyanate (FITC)/propidium iodide (PI). A) Typical flow cytometry pattern of IP6-treated cells using the dot plot with Annexin V-FITC on the X-axis versus PI on the Y-axis. B) rate of apoptotic and necrotic cell death obtained from three independent experiments. *P<0.05 (Student’s *t*-test).

Figure 2 illustrates the rates of apoptotic and necrotic cell death determined using Annexin V-FITC/PI and flow cytometry. No apoptotic cell death of PLB-985 or X-CGD cells was observed following treatment with IP6 for 6 or 24 h. However, IP6 induced necrotic cell death in both cell lines. After 6-h treatment with 20 mM IP6, the rate of necrotic cell death of PLB-985 cells was significantly greater than that of X-CGD cells.

### Inositol hexaphosphate-induced differentiation

**Figure 3:**
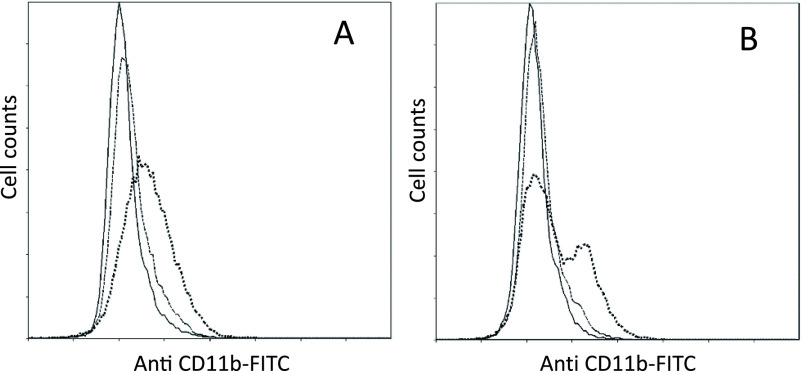
Results of flow cytometry showing inositol hexaphosphate-induced CD11b expression In A) PLB-985 and B) X chromosome linked *gp91-phox* gene knockout cells following incubation with 0 mM (solid line), 5 mM (broken line), and 10 mM (dotted line) inositol hexaphosphate for 72 h. Each histogram shows the CD11b level (measured as signal intensity of antibody fluorescein isothiocyanate) on the X-axis versus cell count on the Y-axis.

Incubation of PLB-985 cells with IP6 for 72 h caused changes in their adhesive properties, and the cells exhibited potential attachment to the culture flask. After a 72-h incubation with IP6, PLB-985 and X-CGD cells were trypsinized and incubated with anti-CD11b FITC. Figure 3 presents the flow-cytometric histograms of FITC intensity of the cells. After treatment with 10 mM IP6, two peaks were observed in the histogram obtained from flow cytometry of X-CGD cells. This indicates that IP6-induced CD11b expression was suppressed in X-CGD cells.

### Effects of inositol hexaphosphate on autophagy

**Figure 4:**
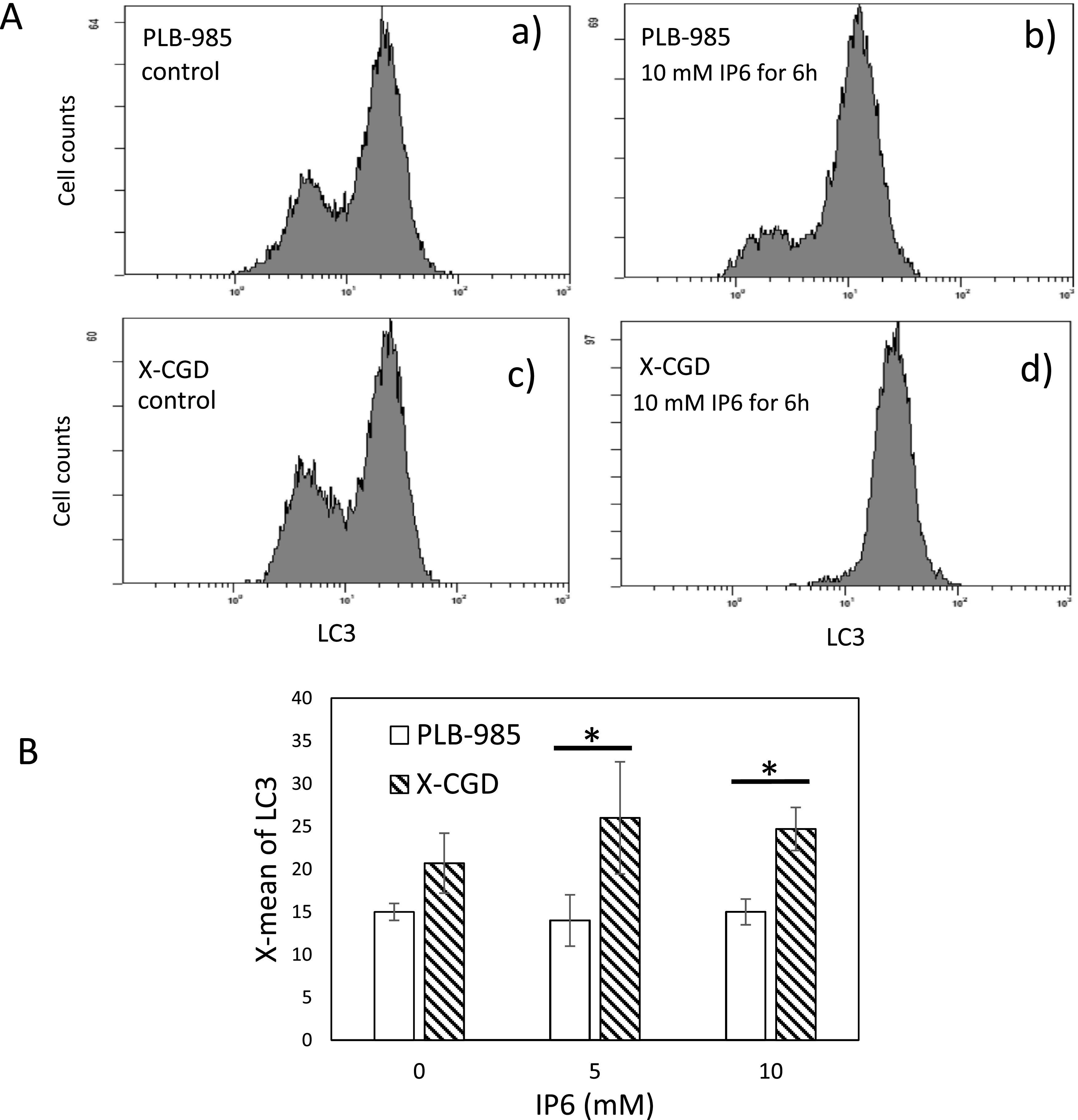
Detection of LC3 levels with flow cytometer A) Typical flow cytometry histogram of cells treated with 10 mM inositol hexaphosphate. Each histogram shows the LC3 levels (measured as signal intensity of the secondary antibody IgG-fluorescein isothiocyanate) on the X-axis versus cell count on the Y-axis. B) The average value (X-mean) of LC3 level calculated from the flow cytometry of the cells treated with 5 and 10 mM inositol hexaphosphate for 6 h obtained from three independent experiments. *P<0.05 (Student’s *t*-test).

The histogram of LC3-FITC in log-phase control cells (prior to IP6 treatment) showed a double peak profile for both cell lines (Figure 4A, a and c). After 6 h of treatment with 10 mM IP6, double peaks were still observed in the histogram of LC3-FITC for PLB-985 cells; however, the intensity of the signal of the main peak was considerably decreased (Figure 4A, b). Nevertheless, the histogram of IP6-treated X-CGD cells showed a single peak, indicating increased LC3 expression (Figure 4A, d). The average value (X-mean) of LC3 calculated from flow cytometry revealed a significant difference between PLB-985 and X-CGD cells (Figure 4B). Our results suggested that the suppression of the bulk degradation system of autophagy resulted in growth inhibition and differentiation in PLB-985 cells.

## Discussion

Understanding the mechanisms that underly IP6-induced biological effects is fundamental to the clinical application of the compound. One of the major benefits of IP6 is its antioxidant effect.^17^ Grases *et al.* reported the IP6 level in plasma to be 0.26±0.03 mg/L in human volunteers who followed a diet that included normal levels of IP6.^32^ Dietary IP6 is believed to protect against intestinal cancer and inflammation.^14^ Norhaizan *et al.* reported that IP6 caused inhibition of cell growth in ovary, breast, and liver cancer cells with 50% maximal inhibitory concentration (IC_50_) values of 3.45, 3.78, and 1.66 mM, respectively.^22^ However, the fact that IP6 ameliorates oxidative stress in biological tissues does not adequately explain the mechanism of IP6-induced cell death. Recent studies investigating the potential mechanisms of IP6-induced biological disorder have suggested that IP6 is associated with the AKT/mTOR signaling pathway, p53 function, NF-kappaB, and the BCL-2 family.^33–36^ The suppression of IP6-induced apoptotic processes through disruption of p53 function in PLB-985 cells, which are p53 null, is considered to be a reason for the low cytotoxicity of IP6 toward PLB-985 cells.^30^

We hypothesized that NADPH oxidase is one of the key components of IP6-induced metabolic disruption. Our experiment involving IP6 treatment of X-CGD cells indicated that cell growth, cell death, differentiation, and autophagy were influenced by knockout of the *gp91-phox* gene. Free IP6 in extracellular fluid functions as an antioxidant through its metal-chelating properties. However, metal-binding IP6 that is incorporated in the phagosome may play a role in amplification of ROS generated by NADPH oxidase. In phagocytes, NADPH oxidase is referred to as NOX2, a homolog of the NOX family that is found in several tissues.^28^ It is widely accepted that ROS generated by members of the NOX family play important roles in a wide range of signal transduction pathways. Our results suggest a novel role of the NOX family as mediators of the biological effects of IP6.
